# Thank You, Edward. Merci, Louis.

**DOI:** 10.1371/journal.ppat.1005320

**Published:** 2016-01-14

**Authors:** Daniel DiMaio

**Affiliations:** 1 Department of Genetics, Yale School of Medicine, New Haven, Connecticut, United States of America; 2 Department of Molecular Biophysics and Biochemistry, Yale University, New Haven, Connecticut, United States of America; 3 Department of Therapeutic Radiology, Yale School of Medicine, New Haven, Connecticut, United States of America; 4 Yale Cancer Center, New Haven, Connecticut, United States of America; University of Michigan Medical School, UNITED STATES

## Smallpox, Less-pox, No-pox

On May 14, 1796, an English country doctor named Edward Jenner launched a new era in medicine. In front of a crowd of curious onlookers, Jenner inoculated eight-year-old James Phipps with pus taken from the sores of a local milkmaid who was afflicted with cowpox, a relatively mild disease. Six weeks later, he challenged the boy with material from a case of the far more serious disease, smallpox. Fortunately for both Phipps and Jenner, the boy did not develop smallpox. Jenner soon inoculated additional subjects, including his own children, with similar results. Vaccination was born.

In the late 18th century, milkmaids often contracted cowpox from their cows, and astute observers noted that milkmaids who recovered from cowpox did not develop smallpox. Although Jenner was not the first to infect people with cowpox in an attempt to ward off smallpox, he conducted the first systematic experiments proving that prior inoculation with cowpox prevented the more serious disease. This was an early example of the scientific method: Jenner formulated his hypothesis and subjected it to rigorous experimental tests. Vaccination proved a safe and effective method to prevent smallpox, but the medical establishment was not immediately impressed. Jenner’s historic study was rejected for publication by the Royal Society and found its way into print as a self-published monograph with a formidable if not particularly informative title, “An Inquiry Into the Causes and Effects of the Variolae Vaccinae: A Disease Discovered in Some of the Western Counties of England, Particularly, Gloucestershire, and Known by the Name of the Cow Pox” [1]. Eventually, Jenner was elected to the Royal Society—not for inventing smallpox vaccination, but in recognition of his observations on the nesting habits of cuckoos. We are fortunate that Jenner’s interests in birds and poxviruses did not lure him into a career studying chicken pox, which was known at the time but has nothing to do with either chickens or poxviruses.

Smallpox was perhaps the greatest killer of all time, one that changed the course of history. Hernando Cortes landed in the Yucatan in 1519 with 500 conquistadors, a few horses, and an invisible army of smallpox viruses that leapt across the front lines and attacked the native population. The Spaniards, who had coexisted with smallpox for centuries in Europe, possessed a measure of protective immunity, but smallpox raged through the immunologically naïve indigenous people and felled them in horrific numbers (up to 90% by some estimates) [2]. The resulting widespread depopulation allowed the rapid European conquest of the Americas. And smallpox continued to rage. In the 20th century alone, smallpox claimed an estimated 300 million lives—many times more than the civilian and military deaths in World War I and World War II combined [2].

Despite its status as arguably the most lethal and feared disease in history, smallpox is also the only human disease ever to be eradicated, a triumph that was made possible by the effectiveness of vaccination and the lack of an animal reservoir. In the 1960s, the World Health Organization mounted a global smallpox vaccination and eradication campaign, which was assisted by the competition between the United States and the Soviet Union to eliminate smallpox from their spheres of influence during the Cold War [3]. This decades-long effort involved sending teams of vaccinators to the most remote regions of the globe and tracking down every case of smallpox on earth. In 1980, the world was declared free of smallpox [4].

The monumental effort to deliver the smallpox vaccine worldwide during the eradication campaign recalled some of the barriers to distributing live cowpox vaccine during the earliest days of vaccination. A major challenge was the transatlantic transport of live vaccine, but the orphans on the docks of Europe provided safe passage. The boys were rounded up and herded on board, and before weighing anchor, one was inoculated with cowpox. When the boy developed cowpox lesions en route, infectious material was taken from these lesions and inoculated into another boy, and so on across the ocean in an unbroken chain of serial arm-to-arm transmission, ensuring that a potent stock of cowpox vaccine arrived in the New World [2]. Necessity is the mother of invention, even if she has to enlist the help of orphans.

## Taking the Bite Out of Rabies

As additional diseases were shown to have an infectious cause, powerful new vaccines were developed. Rabies, polio, yellow fever, measles, and many other viral and bacterial diseases were conquered. The men who developed these vaccines were hailed as heroes—none more so than Louis Pasteur [5]. One of Pasteur’s great achievements was the development of a vaccine against rabies, a disease that is virtually always fatal in humans. In 1885, a dog afflicted with “le hydrophobie” severely mauled a boy, Joseph Meister, who was then brought to Pasteur in Paris. Even though he had tested the vaccine in only a few dogs, Pasteur reluctantly agreed to vaccinate the boy. Meister did not develop rabies, recovered from his injuries, and lived for another 55 years until he took his own life in the face of the Nazi occupation of Paris [6].

We now know that time is an ally in rabies vaccination. After an attack by a rabid animal, the rabies virus must make the long journey up the axons of the peripheral nerves into the central nervous system. During this time, the virus is susceptible to immune attack, so vaccination can prevent rabies even if the vaccine is administered after exposure to the virus.

If you doubt the heroic status granted Pasteur, visit the Institut Pasteur in Paris. You know you are on hallowed ground when you see the life-sized statue of a boy being attacked by a rabid dog. There are monuments to virology elsewhere: One of the outbuildings at the Cold Spring Harbor Laboratory in New York has a roof ornament in the shape of an adenovirus virion, complete with fibers and knobs, and a statue of Edward Jenner sits in London’s Kensington Gardens. But a boy attacked by a mad dog? C’est magnifique!

Inside the Institut Pasteur, you can visit Pasteur’s chambers and his crypt in the basement. There is a marble altar and the stone sarcophagus containing Pasteur’s mortal remains. The walls are covered with inscriptions and garish, multicolored mosaics of allegorical figures and scenes commemorating the great events of Pasteur’s life. You can also visit the room where his scientific apparatus is displayed, including the swan-neck flasks of blown glass, with their long, curved necks to keep bacteria from intruding. In 1859, Pasteur used these vessels to disprove spontaneous generation, Aristotle’s belief that life could arise from nonliving matter [7]. The flasks are surprisingly small: sparrows, not swans. After a century and a half, the broth in the flasks is still clear. How much longer before we can close the books on the experiment?

## Cancer Shots

The story of vaccination did not end with the conquest of acute infectious diseases. Virus infection is now known to be responsible for up to 15% of all cancer deaths, worldwide [8]. Hepatitis B virus (HBV), hepatitis C virus, Epstein-Barr virus, human papillomavirus (HPV), and a handful of other viruses are all human carcinogens. Given the manifest success of vaccination, it seems reasonable to imagine that a vaccine against tumor viruses may prevent some cancers. In fact, a recombinant vaccine against HBV, developed to prevent hepatitis, is lowering the incidence of liver cancer [9]. But could a vaccine be developed whose primary goal is to prevent cancer? Developing and testing such a vaccine poses unique challenges. Most importantly, the great majority of people infected with a tumor virus such as HBV or HPV do not develop cancer, and those cancers that do arise occur years or decades after exposure to the virus. Thus, the ability of a cancer virus vaccine to prevent cancer can be assessed only many years after large-scale vaccination is instituted. And there is a major scientific question: If a single virus particle is sufficient to trigger the multi-year cascade of cellular events that eventually leads to cancer, could a vaccine work well enough to completely block infection and thus prevent the cancer? Despite these challenges, vaccines have been developed that protect against HPV infection, which is responsible for essentially all uterine/cervical cancers and a substantial fraction of other anogenital and oropharyngeal cancers [10]. It is too soon to know whether these vaccines prevent cancer, but there is great optimism that they will because accumulating evidence shows that they can prevent the antecedent precancerous lesions [11–14].

In recent decades, viruses have been postulated to play important etiologic roles in a variety of other chronic human diseases, such as autoimmune diseases, chronic fatigue syndrome, and neurodegenerative diseases. If the association of viruses with these diseases is due to a causal role, vaccination against other intractable chronic diseases may someday become a reality.

## In Defense of Vaccines

First introduced little more than 200 years ago, vaccination is one of medicine’s great achievements. It has prevented countless deaths and untold misery. Based on the pioneering work of Jenner and Pasteur and those that followed, the paths to developing vaccines, and our understanding of their actions, are advanced. It is not always easy, and it would be foolish to think that biology will not throw barriers in our path. After all, an HIV vaccine is still elusive, despite valiant efforts for many years and great expense. Nevertheless, we can now control many infectious diseases through vaccination, and additional vaccines are under development. Unfortunately, for a variety of social, political, religious, and other reasons, some people have rejected vaccination [15]. Although there are certainly rare adverse events associated with some vaccines (e.g., [16]), these are far outweighed by the societal benefits of controlling severe contagious diseases at the population level [15]. There is overwhelming scientific, medical, and public health evidence that vaccines in current use are remarkably safe and effective, and claims to the contrary largely reflect an uninformed position not supported by the facts [15] (e.g., reference [17] presents a convincing refutation of the discredited association between vaccination and autism). Perhaps the problem is that vaccines are too successful, and we have forgotten how terrible these diseases are. Hopefully it won’t take an avian influenza pandemic or a rogue poxvirus outbreak to remind us how much we owe to vaccination. As scientists and physicians, it is our responsibility to persuade people how fortunate we are to live in the vaccine era.
